# Robotic Urologic Surgery in Infants: Results and Complications

**DOI:** 10.3389/fped.2019.00187

**Published:** 2019-05-14

**Authors:** Christina Kim

**Affiliations:** Department of Urology, University of Wisconsin-Madison, Madison, AL, United States

**Keywords:** infants, robotic, pediatric, laparoscopy, outcomes, complications, indications

## Abstract

Over the last 30 years, robotic surgery has evolved into the preferred surgical approach for many operative cases. Robotics has been associated with lower pain scales, shorter hospitalizations, and improved cosmesis (1, 2). However, its acceptance in pediatrics have been hampered by longer operative times, smaller working space, and limited fine surgical instruments. Many find these challenges even more pronounced when performing robotic surgery in infants (i.e., children <1 year old). Although the data in infants is less robust, many studies have shown benefits similar to the adult population. Specifically, multiple reports of robotic surgery in infants have shown lower postoperative analgesic use. Additionally, hospital stays are shorter, which may lead to quicker return to work for parents and guardians. Multiple reports have shown low complication rates of robotic surgery in infants. When complications have occurred, they are usually Clavien Grade 1 and 2, with occasional grade 3. Often the complications are not from the robotic technique, but are linked to other factors such as the ureteral stents (3, 4). Most importantly, the success rates of surgery are comparable to open surgery. This chapter will review indications for the most common urologic robotic surgeries performed in infants. Also, we will review reported results and complications of robotic surgery in children, with specific attention to the infant population. However, data focused only on infants is limited. Many studies have some infant patients, but their results are often mixed with all pediatric patients.

## Background

Initially, urologic interest in minimally invasive surgery was demonstrated in the adult population. In 1993, Kavoussi and Peters described laparoscopic pyeloplasty as an alternative to the open technique. This was in a 24 year old female (5). Moore et al. reported the first 30 adult laparoscopic pyeloplasties at John Hopkins. Mean operative time was 4.5 h. At the time, their postoperative morbidity was low with convalescence of 3 weeks and a mean hospital stay of 3.5 days. Mean follow up of 16.3 months demonstrated radiographic improvement in 97% of the patients (6).

Similar results have been seen when looking at minimally invasive surgery in children. Bauer et al compared outcomes of laparoscopic and open surgery. In 1999, they compared 42 laparoscopic and 35 open pyeloplasties. Pain relief, improved activity levels, and radiographic improvement were similar in these two groups (7). Other series continued to show advantages relative to length of stay (LOS), pain, and cosmesis when comparing laparoscopic and open pyeloplasties (7, 8).

When robotic surgery was introduced, there was excitement due to three-dimensional imaging, 10-fold camera magnification, tremor filtering, and new camera control by the surgeon. Also, there is instrument articulation with full range of motion. Surgeons hoped these enhancements would allow precise suturing, improve tissue handling, and increase ease of doing complex surgical cases. Robotics quickly gained popularity in adult urology for prostate surgery (9, 10). In 1995, Partin et al. described a variety of robotic procedures in 17 adult patients. They saw 3 minor complications and no significant difference in operative time. They were encouraged by the feasibility of robotic surgery, but encouraged more data collection to assess safety and efficacy of robotic surgery (11).

Similar to the adult literature, pediatric robotic surgery has been associated with lower pain scales, less narcotic use, and shorter hospital stays (12).

## Cosmesis of Robotic Surgery

From a cosmetic standpoint, satisfaction cannot be self-assessed by an infant. But Barbosa et al. looked at parental and non-infant patient preference regarding scar appearance. Three groups were presented comparing scars with open and robotic pyeloplasties, ureteral reimplantation, and bladder augmentation. For patients under the age of 7 years, their parents were also asked to fill out the survey. One hundred and sixteen parents and 19 patients filled out the surveys. For pyeloplasty, the most important factors were scar visibility, scar size, scar location, and ability to cover the scar. These results support the concept that smaller scars associated with robotic surgery is preferred by patients and parents. However, the preference was less marked in the pyeloplasty group. And the most common urologic infant robotic surgery is pyeloplasty (13).

Casale supports robotic surgery in infants due to equivalent surgical outcomes with robotics with negligible external scars and less manipulation of tissue. There is less collagen deposition with robotic incisions when compared to open surgery. So scarring from smaller robotic ports may be advantageous not only from aesthetics, but also from tissue healing (14).

When comparing one longer open incision to multiple small robotic ports, studies have shown less total tension across non-linear wounds, compared to a longer single incision. Pathologic scarring is consistently greater with high tension wounds (e.g., keloids) (15). Therefore, smaller trocar incisions should minimize pain and associated scarring (16).

## Pain with Robotic Surgery

Looking at pain management with robotic surgery in children, there is a paucity of data. Many of the physiologic effects of robotic surgery are well-understood. Due to increased intra-abdominal pressure, there is decreased lung compliance, decreased functional residual capacity, and increased airway resistance. When pressures are high enough, there can be increased cardiac output with peripheral vasoconstriction. Also, there can be increased renovascular resistance and decreased flow through the renal vein (17). However, there is little data reviewing the anesthetic considerations and postoperative management for robotic surgery in children.

Most reports on pain simply summarize narcotic use. Tanaka et al. compared open and laparoscopic pyeloplasties and saw a lower narcotic charge in pre-adolescent and adolescent populations. However, they did not see a difference in patients under the age of 10 years (18). Smith et al. compared open and robotic reimplantation in 50 patients ranging 3–144 months old. When compared to open repairs, they saw lower narcotic use in the bilateral robotic reimplantation. However, they did not see a statistically significant difference when comparing the unilateral reimplantations. Also, they did not outline the use of non-narcotic analgesics (19).

Some reports assessed both narcotic use and pain scales. Marchini et al. did a retrospective review of open and robotic reimplantation where pain scales and narcotic use were summarized. Although there was no significant difference in pain for the two cohorts, their analysis only included patients with pain scales >2 (20). Lee et al. compared results between an open and laparoscopic cohort. They did not see a statistically significant difference in pain scales for patients <2 years old (21). Harel et al. did a prospective review comparing pain assessment after robotic and open ureteral reimplantation. Amongst 34 patients, 11 patients had open surgery and 23 patients had robotic surgery. Robotic patients had lower narcotic requirement and lower intensity of postoperative pain. Although there was no significant difference between the two groups' subjective pain scores, the open cohort had more severe pain (45 vs. 9%). However, this was a small sample size that lacked randomization. And similar to the other studies described, this experience was not limited to an infant population (12).

## Costs of Robotic Surgery

It is challenging to compare costs of surgery. The “comparative cost” of surgery is the quality divided by cost. Quality includes many factors (e.g., effectiveness, safety, patient satisfaction). And data summarizing these factors is still limited in both adult and pediatric patients. In 2009, a KID database comparison showed a $2,500 advantage with open pyeloplasties (22). In 2010, Varda et al showed a $3,500 discrepancy favoring open pyeloplasty (23).

As stated earlier, the use of robotics continues to increase each year. The increased experience should drive down operative times. As operative times decrease, operative costs decrease as well. Also, the more experienced robotic surgeons may lower costs with their judicious use of disposable instruments (24).

Some studies omit the amortization cost of the device, as well as the cost of maintenance for the machine. Many reports simply summarize the cost of the instruments used for that specific case. However, the estimated additional cost per case from amortization is $1,600 (25). Dangle et al. did not see a significant difference in direct cost of robotic and open pyeloplasties (4). Casella et al. compared robotic and laparoscopic pyeloplasties. There were 23 patients in each group. There was no significant difference in cost between the two groups. When looking at a subgroup of robotic cases where the stent was placed antegrade, they did see decreased operative time and lower total costs (*p* < 0.001) (24).

Varda et al. recently reviewed national trends in pediatric pyeloplasties. This analysis reviewed 2003–2015. Although the overall total volume of pyeloplasties has decreased by 7%, the number of robotically-assisted pyeloplasties has risen by 29% annually. There were few open cases in adolescents and few robotic cases in infants. They tried to analyze the adjusted outcomes and median costs for open and robotic pyeloplasties. Also, they tried to determine the primary drivers of cost for both open and robotic pyeloplasties. The three primary cost contributors were: operating room (OR) cost, equipment costs, and room/board. Room/board costs were higher for the open cases, whereas OR and equipment costs were higher for robotic cases. They found a higher median cost with robotic cases, and an absolute difference in cost per case of $1,060 (26).

There are many quality factors that may still favor robotic surgery (e.g., cosmesis, patient satisfaction, parental return to work). Improvement in these factors can improve the hospital's reputation. This may expand referral patterns not only for pediatric urology, but also for other departments in the hospital. Therefore, the cost and value of robotic surgery for a pediatric hospital is complex and nuanced.

## Technical Challenges of Infant Robotic Surgery

One potential barrier to robotic surgery in infants is robotic arm collisions due to the small working space. Finkelstein et al looked at 45 robotic cases performed in infants 3–12 months old. They looked at console time and number of robotic collisions. They found less collisions when the anterior superior iliac spine measurement (ASIS) of >13 cm or puboxyphoid distance (PXD) of >15 mm. These results were from a single surgeon, so it may not be translatable to the masses (27).

In regards to proficiency in technique, laparoscopic intracorporeal suturing is cumbersome and technically challenging for many surgeons. These technical challenges hampered enthusiasm for standard laparoscopic pyeloplasties and ureteral reimplantations. However, robotics offered more precise and efficient suturing (21). This widened the utilization of minimally invasive surgery for not only ureteropelvic junction obstruction (UPJO) and vesicoureteral reflux (VUR), but also for partial nephrectomy, bladder augmentation, and creation of catheterizable stomas.

In contrast to standard laparoscopy, robotic surgery has a quicker learning curve (28). Sorenson et al. compared the first 33 robotic and open pyeloplasties performed by senior faculty. When comparing the groups, there was no significant difference in length of stay, pain score, or surgical success. The number of complications were identical in the two groups. However, after the first 15–20 robotic cases, overall robotic operative times were within one standard deviation of the open pyeloplasty (29). Dangle et al. found their operative time decreased by 20 min after their first 5 robotic cases (4). Lee et al. compared outcomes with open (OPN) and robotic (RALP) pyeloplasties. Linear regression and ANOVA showed no significant change in time for the OPN group, but there was significant improvement in the RALP group (21). Kassite et al. analyzed the learning curve for two surgeons who were new to performing robotic surgery. A total of 42 RALP were performed in 41 patients. They accounted for patient complexity factors. Not surprisingly, they found that complexity factors influenced surgical outcomes. After looking at patient complexity factors and the perioperative data, they felt that more than 41 cases are needed to achieve mastery (30).

## Robotic Case Selection

Over time, data has grown in pediatric robotic surgery. In other disciplines, pediatric robotic surgery has been used for a wide variety of surgical cases. The majority of these cases have been abdominal, but some have been thoracic cases. In 2008, Meehan reported 24 different robotic procedures in children. The majority of these cases had never been done with a minimally invasive approach by these specific authors. In this series, the only conversions were due to equipment failures or issues with standard laparoscopic equipment through the robotic ports. But no conversions were due to injuries from robotic instruments. They felt nursing team was critical to positive outcomes. In an effort to strengthen central organization, their hospital appointed a scrub nurse as their robotic coordinator. They streamlined training for the circulating nurse. They felt the designated personnel improved their set up and turnover time. Once in place, all technical aspects of robotic cases improved (31).

Although the data of robotic surgery in infants is less robust, results have shown benefits similar to the adult population. Reports in infants have shown lower postoperative analgesic use. Also, hospital stays are shorter, which may lead to quicker return to work for parents and guardians (4, 32, 33).

Multiple reports have shown low complication rates of robotic surgery in children. When complications have occurred, they are usually Clavien Grade 1 and 2. Often the complications are not from the robotic technique, but are linked to the ureteral stents (3, 4, 34). Most importantly, the success rates of surgery are comparable to open surgery.

A wide variety of robotic procedures have been described in children. However, reported outcomes in children (particularly in infants) are limited. To date, pyeloplasty is the primary pediatric robotic surgery with comparable safety and efficacy when compared to open or standard laparoscopic approach. This has been supported by large multi-centric studies (35). Also, this has been supported by the European Association of Urology Pediatric guidelines. The guidelines recognize the benefits of minimally invasive surgery by stating that “in experienced hands, laparoscopic or retroperitoneoscopic techniques and robot-assisted techniques have the same success rates as standard open procedures.” Also, they state that “Robotic-assisted laparoscopic pyeloplasty has all the same advantages as laparoscopic pyeloplasty plus better maneuverability, improved vision, ease in suturing and increased ergonomics but higher costs.” However, the role for robotic pyeloplasty in infants is less supported when the EUA states “There does not seem to be any clear benefit of minimal invasive procedures in a very young child, but current data is insufficient to defer a cut-off age (36).”

Laparoscopic and robotic ureteral reimplantation have not been widely accepted due to longer operative times and varied success rates. Many reports show success rates with robotic reimplantation lower than open repairs (37–39). And anti-reflux surgery is rarely indicated in infancy.

Robotic partial nephrectomy has been described in children and in some infants. Some find the dexterity and visualization of vascularity superior with robotics. Many find suturing more efficient with robotics. This is pertinent when buttressing sutures are placed in the remaining healthy renal tissue. Also, suturing may be required in the collecting system.

Given the increased dexterity, robotics can be helpful when performing complex reconstruction (e.g., bladder augmentation, Mitrofanoff creation, bladder neck reconstruction). This was first described in 2002 (40). Robotic bladder augmentation have longer operative times than open surgery, but also have lower blood loss and shorter hospital stays (41, 42). However, these complex reconstructive cases represent a small part of the existing literature in pediatric robotic surgery (43–45). And these complicated reconstructive cases are not done in infancy.

Although a wide variety of pediatric urologic cases have been performed with robotic assistance, its primary indication in pediatrics is for robotic pyeloplasty.

## Case Specific Surgical Outcomes

### Pyeloplasty Surgical Outcomes

As stated earlier, the most commonly performed urologic robotic surgery is a pyeloplasty for a ureteropelvic junction obstruction (UPJO). For many years, open pyeloplasty has been considered the gold standard for therapy (36). Many early reports on pediatric robotic-assisted laparoscopic pyeloplasties (RALP) compared results with laparoscopic and open techniques. However, these were small, single center case series on 10 or fewer patients (32, 46, 47).

In 2006, Lee et al. compared robot assisted laparoscopic dismembered pyeloplasty (RALP) to an age matched cohort of patients undergoing open pyeloplasties (OPN). There were 33 patients in each cohort. In this series RALP was safe and effective. Thirty-one of the 35 RALP had improvement in radiographic follow up and/or symptoms. Their LOS was shorter (2.3 vs. 3.5 days). RALP patients had higher intraoperative narcotic use. But use of epidurals was vastly different. Eighteen OPN patients had an epidural and no RALP patients had an epidural. Overall, the RALP patients had lower postoperative and total narcotic use (*p* = 0.001). Also, linear regression analysis showed a longer LOS in the OPN group as age of patient increased. However, there was no difference in LOS for the RALP group. There was similar estimated blood loss (EBL) in both cohorts. And no blood transfusions were required for either group. Mean operative time was higher in the RALP group (219 vs. 181 min). But this was not statistically significant (*p* = 0.031). There were no complications in the OPN group. One patient from the RALP group required repeat surgery. This patient initially had a retroperitoneal surgery and crossing vessels were not recognized. Due to persistent obstruction, this patient had a temporary percutaneous nephrostomy tube and later had a transperitoneal repair. Follow up in this series was short, with a mean follow up of 10 months for the RALP cohort. Similar to other studies, increased experience correlated with quicker operative times (21).

When looking specifically at results in infants, the data is less robust. Ballouhey et al. evaluated robotic surgical results in patients under and over 15 kg. They found success rates were comparable. They had 62 patients with a mean weight of 11.1 kg and 116 patients with a mean weight of 30.2 kg. The mean follow up was 37 months. The most common surgeries were pyeloplasty, nephrectomy, and fundoplication. Although set up time was longer in the smaller patients, the overall surgical time and hospital stays were not statistically different (48).

Kutikov et al. had one of the earliest reports of robotic surgery in infants. They did a retrospective review of robotic pyeloplasties in 9 infants aged 3–8 months. Mean operative time was 122. Eight minutes with a mean console time of 72.1 min. The mean hospital stay was 1.4 days. Seventy-eight percent had resolution or improvement in their hydronephrosis. No patient required conversion to open or standard laparoscopic techniques (32).

Kawal et al. looked at their 4 year experience of robotic pyeloplasties in 138 patients, 34 of whom were infants. In their series, multivariate and comparative analysis showed lower morphine equivalents in infants. Of note, infants had a higher chance of placing a percutaneous stent. The infant cohort had success rates of 96%. Six patients (4%) required repeat surgery. Although infants had a 29.4% complication rate, this was similar to the older population (30.8%). Reported complications were low grade: 60% were Clavien grade 1 and 2 (pain, urinary tract infection). Forty percent were Clavien grade 3 (stent dislodgement and replacement). The most common complications with both infants and older children were stent related, with evaluation in the emergency room for pain and hematuria (49).

Dangle et al. reviewed their experience with infant pyeloplasties comparing open and robotic approaches. They had 10 patients in each arm. Mean patient age was 3.31 months. Postoperative outcomes were similar in for the open vs. robotic arms: length of stay (2.2 vs. 2.1 days), estimated blood loss (6.5 vs. 7.6 ml), days to regular diet (1 vs. 1.1 days), and time to foley removal (1.3 vs. 1.3 days). However, total operating time was longer in the robotic group (199 vs. 242 min). When excluding amortization, robotic cost, maintenance and depreciation, direct costs were similar ($4,410 vs. $4,979 per case). In regards to surgical success, improvement in hydronephrosis was identical in both groups. These authors recognize the importance of surgeon experience before performing robotic surgery infants. Their senior author had performed 28 pyeloplasties and 60 other complex robotic procedures in older children before forging into robotic surgery in infants (4).

In 2015, Avery et al. reported a multi-institutional experience of infant robotic pyeloplasty. They reported the results by 6 surgeons at 5 different institutions. Sixty patients under the age of 12 months underwent 62 robotic pyeloplasties. All patients had this done with a transperitoneal approach. Mean age was 7.3 months and mean weight was 8.1 kg. There was a 91% success rate with an 11% complication rate. The complications were Grade 1 (1 patient), Grade 2 (2 patients), and Grade 3 (4 patients). No visceral or vascular injuries occurred. But the complications included: two port hernias, one urine leak, one retained stent, one ileus, one renal calculus, and one urinary tract infection. Seventy-two percent were discharged on postoperative day 1. All six surgeons did have more than 5 years of experience post fellowship training (3).

Since 5 mm robotic instruments have a longer articulating arm, some surgeons steer away from using the 5 mm instruments, in favor of 8 mm instruments. Baek et al. compared the perioperative parameters for infant and non-infant RALP over a 2 year period of time. There were 16 infants and 49 non-infants. There was no difference in operative time, hospital pain medication use, or hospital stay. Success rates were similar: 93% for infants and 100% or non-infants (*p* = 0.08) (50).

### Ureteral Reimplantation Surgical Outcomes

Outcomes with robotic ureteral reimplantation are still evolving. And the data is very limited for infants. Initial robotic experience entailed an intravesical approach. This approach had varied success rates between 83 and 100%. And complication rates were 0–52% (20, 51, 52).

There is more data with robotic ureteral reimplantation surgery performed with an extravesical approach. In these series, complication rates have ranged from 0 to 40%. However, success rates have varied between 77 and 100%. Robotic ureteral reimplantation has not become a standard of care for anti-reflux surgery (4, 21, 37, 38, 53–55).

However, in many robotic ureteral reimplantation series, the youngest patients are still over a year old. Herz et al. reported their experience with extravesical ureteral reimplantation in 72 ureters (54 patients). They had success in 84.7%, but the youngest patient was 2.5 years old (37). Chalmers et al reported their results in 17 patients with a 90.9% success. However, all patients were over 2 years old (55). Dangle et al. reported on 29 patients with a success rate of 87.5% but the youngest patient was 3 years old (56). Grimsby et al. reported the combined experience from two institutions with 93 ureters treated in 61 patients. Although some patients were under 1 year, the mean age was 6.7 years. Their success rate was 72%. Boysen et al. showed improved results in a prospective multi-institutional study of extravesical reimplantation. They reported from 7 institutions treating 143 patients (199 ureters). Success rate was 93.8%. Mean age was 6.6 years (57). Akhavan et al. reported on 78 ureteral reimplantations performed at their institution. Success rates were good with only 7.7% of patients with persistent reflux. Also, there was 10% complication rate. However, the mean age was 6.2 years old with the youngest patient 1.9 years old. Although the authors felt RALUR was effective and safe treatment for primary vesicoureteral reflux, it was not an experience for infant patients (53).

In 2011, Smith et al. described an infant extravesical robotic reimplantation on a 3 month old infant (19).

Complication rates with robotic ureteral reimplantations have been low. Boysen et al. did a multi-institutional review from nine institutions. This included a total of 260 patients (363 ureters). The overall complication rate was 9.6%. There were no Clavien Grade 4 or 5 complications. This was a large cohort, but not specific to infants (58).

When indicated, performing a ureteral reimplantation has restricted use in infants due to their small bladder capacity. With an intravesical approach a bladder capacity of 130 mL is preferred (59). Given the limited data and rare need for intervention in infancy, there is no defined role for robotic ureteral reimplantation surgery in infants.

### Miscellaneous Cases

Ballouhey et al. looked at robotic partial nephrectomy in small children. This was not specific to infants, but it was a cohort of 28 patients all <15 kg: 15 patients done with a robotic approach and 13 patients done with an open approach. Mean at the time of surgery was 20.2 months for the robotic arm and 18.4 months for the open arm. The mean hospital stay was significantly longer for the open arm (6.3 vs. 3.4 days) *P* < 0.001. Also, the postoperative pain control in total morphine equivalent intake was significantly greater in the open arm (1.08 vs. 0.52 mg/kg/day) *P* < 0.001. There was no significant difference in terms of operating time, complication rate, or renal outcomes (60).

Robotic partial nephrectomy has been demonstrated in infants. Wietsma et al. described their experience doing a robotic lower pole partial nephrectomy in an 11 month old male who was 10.7 kg. This patient had no intraoperative or postoperative complications. He was discharged home on postoperative day 1 (61). However, the experience in infants is still limited, so it is not a standard of care.

Bansal et al. described a bilateral upper tract robotic surgery in a 4 month old infant (left ureteroureterostomy and Right upper pole partial nephrectomy). There were no intraoperative complications and the patient went home on postoperative day 1 (62). They expanded their review of 10 infants who underwent robotic upper tract reconstructive surgery at their institution between March 2009 and February 2013. Eight patients underwent pyeloplasty and 2 underwent ureteroureterostomy. The mean age was 10 months and mean weight was 7.7 kg. Mean follow up was 10 months. Postoperative ultrasound showed improved in all patients. There were three complications (one Grade 1 and two Grade IIIb). Complications included ileus, urinary tract infection, and one urine leak (63).

Looking a broader view of robotic cases, Fuchs et al. did a retrospective review of multiple upper tract surgeries done in infants. A total of 67 patients had surgery: 26 pyeloplasties, 18 heminephrectomies, and 23 nephrectomies. Mean weight was 6.4 kg and mean operative time was 113 min. One pyeloplasty required conversion to open technique. One patient had a missed intraoperative bowel injury. No blood transfusions were required. The pyeloplasties had improvement in their drainage time. And the heminephrectomy patients had stable postoperative renal function. This group preferred a transperitoneal approach due to the size limitations in infants (64).

Srougi et al. looked at their institution's experience doing robotic surgery in infants and toddlers. However, of the 65 patients in their series, only 14 patients were infants under the age of 1 year. There was a wide range of cases performed (pyeloplasty, nephrectomy, reimplantation, ureteroureterostomy, orchidopexy, excision of Mullerian remnant, and pyelolithotomy). Mean hospital stay was 1.3 days. Mean weight was 11.6 kg, but they did evaluate the complication rate in children <10 kg. They had 23 patients under 10 kg (34%). There were 12 post-operative complications. Most were Clavien grade I and II. But there was one grade IIIB complication. There was not a higher complication rates in the smaller children. In fact, the patients >10 kg had higher complication rates, but it was not statistically significant (65).

Feasibility of performing robotic surgery in infants has been shown in other fields. Meehan et al. reported on 45 infants who underwent robotic surgery. Eighty-nine percent of the patients had surgery completed with a robotic technique. The average age was 8 months and average weight was 6.8 kg (66). Dawrant et al. described their experience doing robotic-assisted resection of choledochal cysts and hepaticojejunostomy in infants <10 kg. In 2009, they performed this surgery in 5 children. Mean age was 1 year and mean weight was 8.5 kg. Mean discharge was on postoperative day 6. There were no postoperative complications (67).

Overall complication rates of robotic surgery in children has been reportedly low. Bansal et al. looked the complication rate by 3 surgeons during the first 4 years of their robotic program. This review included 10 infants, but was primarily non-infant pediatric cases. Ten different surgeries were performed in 136 patients. Only one of the surgeons performed surgery on infants. They were all performed transperitoneally. There were no intraoperative complications, robotic malfunctions or conversions to open surgery. Eleven patients experienced a postoperative complication. Three of these 11 complications occurred in infants. Therefore, the complication rate for infants was 30% (3 out of 10) and 8.6% for the other pediatric patients (8 out of 126 non-infants) *p* = 0.035. There were 2 Clavien grade 1, 7 Clavien grade II, and 2 Clavien grade IIIb. The degree of complications was not higher in the infant patients. And none of the complications were due to intraoperative or due to robotic malfunction (34).

## Conclusion

Robotic surgery continues to evolve in pediatric urology. There are multiple series demonstrating excellent surgical results. The benefits of shorter hospital stays, less narcotic use, improved cosmesis has been demonstrated in both adult and pediatric populations. However, there remains limited data on robotic surgery in infants.

Robotic surgery in pediatrics had steadily gained acceptance, but there are many surgeons still hesitant to utilize this technique in infants. Concerns include the limited operative space, relatively large port sizes, increased operative time, and potential decreased anesthesia access to the patient (68, 69). National trends in pediatric pyeloplasties have remained fairly stable. The volume of robotic repairs has increased and the number of open repairs has decreased. However, a review of national trends showed that infants were 40 times less likely to have a robotic repair when compared to older children (23).

However, hesitancy to use robotics in infants and children may be misguided. There are many reports of robotic surgery that confirm it is a safe and feasible technique. Although it has been used for a wide variety of urologic cases, its primary indication is limited to pyeloplasty. Many reports demonstrate comparable results of robotic pyeloplasty relative to open surgery. Robotic partial nephrectomy has also been shown safe and effective, albeit in modest data.

More data is needed, but robotics is a safe and effective approach for a wide array of urologic cases, even in infants.

## Author Contributions

The author confirms being the sole contributor of this work and has approved it for publication.

### Conflict of Interest Statement

The author declares that the research was conducted in the absence of any commercial or financial relationships that could be construed as a potential conflict of interest.
